# Diastolic blood pressure predicts enlarged vertebral venous plexus and intracranial pressure in patients with bilateral transverse sinus stenosis

**DOI:** 10.3389/fneur.2022.957353

**Published:** 2022-08-22

**Authors:** Min Li, Xiaogang Gao, Fengwei Liu, Jingkun Sun, Ning Xia, Ran Meng, Xunming Ji

**Affiliations:** ^1^Department of Neurology, Xuanwu Hospital, Capital Medical University, Beijing, China; ^2^Beijing Institute for Brain Disorders, Capital Medical University, Beijing, China; ^3^Department of Medicine, Tianjin Huanhu Hospital, Tianjin, China; ^4^Department of Neurology, Massachusetts General Hospital, Boston, MA, United States; ^5^Department of Neurosurgery, Xuanwu Hospital, Capital Medical University, Beijing, China

**Keywords:** diastolic blood pressure, intracranial pressure, enlarged vertebral venous plexus, transverse sinus stenosis, venous sinus stenosis

## Abstract

**Background:**

Bilateral transverse sinus stenosis (BTSS) is associated with intracranial hypertension. Enlarged vertebral venous plexus (EVVP) refers to a compensation mechanism against elevated intracranial pressure (ICP) in patients with BTSS. This study aims to investigate the influencing factors of EVVP.

**Methods:**

Patients with BTSS were prospectively recruited from the neurology department and neurosurgery department of Xuanwu Hospital Capital Medical University from January 2020 to December 2021.

**Results:**

A total of 37 patients were enrolled with a mean age of 45.42 ± 15.64 years. Women tend to be more susceptible to BTSS. The most common co-morbid disease was hypertension. The most common clinical manifestations were visual disorders, headaches, and tinnitus. BMI and DBP were significantly higher in BTSS patients without EVVP than those with EVVP. Multivariate analysis revealed that diastolic blood pressure (DBP) was negatively correlated with EVVP. In addition, a positive correlation between DBP and the ICP was also observed. A DBP of 81.5 mmHg was calculated as the cutoff value for the presence of EVVP. BTSS patients with DBP ≤ 81.5 mmHg had a higher incidence of EVVP and a lower ICP compared to those with DBP > 81.5 mmHg.

**Conclusions:**

DBP was identified as an independent predictor of EVVP. DBP was lower (≤81.5 mmHg) in patients with EVVP and therefore was associated with a lower ICP in patients with BTSS.

## Introduction

Transverse sinus stenosis is known as the narrowing of the unilateral or bilateral transverse sinus, with the prevalence of unilateral TSS and bilateral TSS (BTSS) in the general population of 33% and 5%, respectively (1). However, cases of symptomatic unilateral TSS are rarely reported (2); contrastively, BTSS is deemed as an independent cause of intracranial hypertension (IH), bringing about clinical symptoms, such as headache, tinnitus, and blurred vision (3, 4). Nonetheless, it is noteworthy that not every patient with BTSS experiences IH (5). Our previous study reported that the presence of enlarged vertebral venous plexus (EVVP) compensates for the elevated intracranial pressure (ICP) in patients with BTSS (6). However, no previous study has reported any influencing factor of EVVP. This study aims to find out the influencing factors of EVVP.

## Materials and methods

### Patient recruitment

In this study, we prospectively recruited patients with BTSS who were admitted to the neurology department and neurosurgery department at Xuanwu Hospital Capital Medical University from January 2020 to December 2021. The inclusion criteria were as follows: symptomatic cases diagnosed as BTSS by magnetic resonance venography (MRV) and/or digital subtraction angiography (DSA). The representative images of BTSS and EVVP were as previously mentioned (6). The exclusion criteria were defined as follows: patients with (1) drug-related IH; (2) brain parenchymal lesions; (3) moderate to severe stenosis in intracranial, carotid, or vertebral arteries, (3) moderate to severe stenosis in internal jugular veins or intracranial sinuses other than transverse sinus. All patients signed the informed consent, and their anonymity was preserved. This study was approved by the Xuanwu Hospital ethics committee.

### Data collection

Since ICP measured by lumbar puncture agreed well with that measured intracranially (7), lumbar puncture was performed in the morning of the 2nd day of hospitalization to obtain the ICP of each patient. Age, gender, body mass index (BMI), past medical history, personal history, vital signs at admission, clinical manifestations, neuroimaging, and ICP were recorded.

### Statistical analysis

All statistical analyses were conducted using SPSS Version 16 (SPSS, Inc., Chicago, Illinois, United States). Continuous data were expressed as mean ± standard deviation (SD) and processed by using student's *t*-test. Categorical data were reported as numbers (percentage) and processed by using the chi-square test. Linear regression was used to predict the relationship between two variables. Binary logistic regression analysis was conducted with the enter method to identify the odds ratio (OR) and the corresponding 95% CI. A cutoff point was calculated by using receiver operating characteristics (ROC) curves. A *p*-value of **<**0.05 was considered statistically significant.

## Results

A total of 37 eligible patients with BTSS were included in this study, with a mean age of 45.42 ± 15.64 years and an average body mass index (BMI) of 25.47 ± 4.26. A gender-specific prevalence of BTSS in women was 83.8%. The most common co-morbid disease was hypertension (29.7%). The smoking rate (5.4%), and alcohol rate (2.7%) in patients with BTSS were apparently lower than those in the general population in China (8). The vital signs were within the normal ranges. Moreover, visual disorder (62.2%), headache (56.8%), and tinnitus (37.8%) were the top three clinical manifestations of BTSS (Table 1).

**Table 1:** Demographic features of the BTSS patients.

**Characteristics**	**Patients with BTSS (*n* = 37)**	**(%)**
Age (years)	45.42 ± 15.64	NA
Female	31	83.8
BMI	25.47 ± 4.26	NA
**Past medical history**		
Hypertension	11	29.7
Diabetes mellitus	0	0
Coronary artery disease	1	2.7
Hyperlipidemia	7	19.5
Hyperuricemia	2	5.4
**Personal history**		
Smoking	2	5.4
Alcohol	1	2.7
**Vital signs at admission**		
Systolic pressure (mmHg)	129.77 ± 12.60	NA
Diastolic pressure (mmHg)	81.26 ± 9.26	NA
Heart rate (bpm)	78.23 ± 10.63	NA
Respiratory rate (/min)	18.97 ± 1.78	NA
Body temperature (°C)	36.45 ± 0.27	NA
**Clinical manifestations**		
Tinnitus	14	37.8
Tinnitus cerebri	11	29.7
Headache	21	56.8
Dizziness	10	27.0
Neck discomfort	5	13.5
Sleep disturbance	7	18.9
Hearing disorder	7	18.9
Visual disorder	23	62.2
Subjective memory decline	5	13.5

BMI, body mass index; BTSS, bilateral transverse sinus stenosis; NA, not available.

Demographic characteristics of patients with BTSS with or without EVVP were summarized in Table 2. BMI (*p* = 0.011) and DBP (*p* = 0.01) were significantly higher in patients with BTSS without EVVP than those with EVVP (Table 2).

**Table 2:** The demographic characteristics of the BTSS patients with or without EVVP.

**Characteristics**	**BTSS patients with EVVP** **(*n* = 18)**	**BTSS patients without EVVP** **(*n* = 19)**	***p***
Age (years)	48.44 ± 17.59	42.20 ± 13.07	0.274
Female (*n*, %)	15 (83.33)	16 (84.21)	0.942
BMI	23.65 ± 4.09	27.42 ± 4.86	0.011^*^
**Co-morbid disease (*n*, %)**			
Hypertension	5 (27.78)	6 (31.58)	0.800
Diabetes mellitus	0	0	NA
Coronary artery disease	0 (0)	1 (5.26)	1.000
Hyperlipidemia	3 (16.67)	4 (21.05)	1.000
Hyperuricemia	1 (5.56)	1 (5.26)	1.000
**Personal history (*n*, %)**			
Smoking	0 (0)	2(10.53)	0.486
Alcohol	0 (0)	1 (5.26)	1.000
**Vital signs at admission**			
Systolic pressure (mmHg)	125.50 ± 6.92	134.33 ± 15.68	0.059
Diastolic pressure (mmHg)	77.25 ± 8.77	85.53 ± 7.95	0.010^*^
Heart rate (bpm)	76.50 ± 10.62	80.07 ± 10.68	0.359
Respiratory rate (/min)	18.75 ± 2.05	19.20 ± 1.47	0.491
Body temperature (°C)	36.41 ± 0.27	36.50 ± 0.27	0.382

* p < 0.05. BMI, body mass index; BTSS, bilateral transverse sinus stenosis; EVVP, enlarged vertebral venous plexus; NA, not available.

Univariate logistic regression analysis showed that BMI (OR = 0.785, *p* =0.014) and DBP (OR = 0.873, *p* = 0.005) were negatively correlated with EVVP (Table 3). Whereas, multivariate logistic regression analysis revealed that only DBP correlated with EVVP (OR = 0.891, *p* = 0.021, Table 3).

**Table 3:** Univariate logistic regression analysis showed that BMI and DBP negatively correlated with EVVP.

**Independent variables**	**Dependent variables**	**OR**	**SE**	***P***	**95% CI**	
					**Lower**	**Upper**
**Univariate logistic regression**						
BMI	EVVP	0.785	0.099	0.014^*^	0.647	0.953
DBP		0.873	0.048	0.005^*^	0.795	0.960
**Multivariate logistic regression**						
BMI	EVVP	0.869	0.095	0.137	0.721	1.046
DBP		0.891	0.050	0.021^*^	0.808	0.982

Multivariate logistic regression analysis revealed that only DBP correlated with EVVP.

* p < 0.05. BMI, body mass index; CI, confidence interval; DBP, diastolic blood pressure; EVVP, enlarged vertebral venous plexus; OR, odds ratio; SE, standard error.

Subsequently, the impact of BMI and DBP on ICP was investigated. Univariate linear regression analysis showed that BMI (Coefficient = 6.451, *p* = 0.049, Table 4) and DBP (Coefficient = 3.968, *p* = 0.01, Table 4) positively correlated with ICP. However, multivariate linear regression analysis revealed that only DBP (Coefficient = 3.305, *p* = 0.04, Table 4) correlated with ICP.

**Table 4:** Univariate linear regression analysis showed that BMI and DBP positively correlated with ICP.

**Independent variables**	**Dependent variables**	**Coefficient**	**SE**	***P***	**95% CI**	
					**Lower**	**Upper**
**Univariate linear regression**						
BMI	ICP	6.451	3.155	0.049^*^	0.015	12.886
DBP		3.968	1.443	0.010^*^	1.018	6.918
**Multivariate linear regression**						
BMI	ICP	4.017	3.330	0.238	−2.805	10.839
DBP		3.305	1.533	0.040^*^	0.164	6.446

Multivariate linear regression analysis revealed that only DBP correlated with ICP.

* p < 0.05. BMI, body mass index; CI, confidence interval; DBP, diastolic blood pressure; ICP, intracranial pressure; SE, standard error.

**Figure 1:**
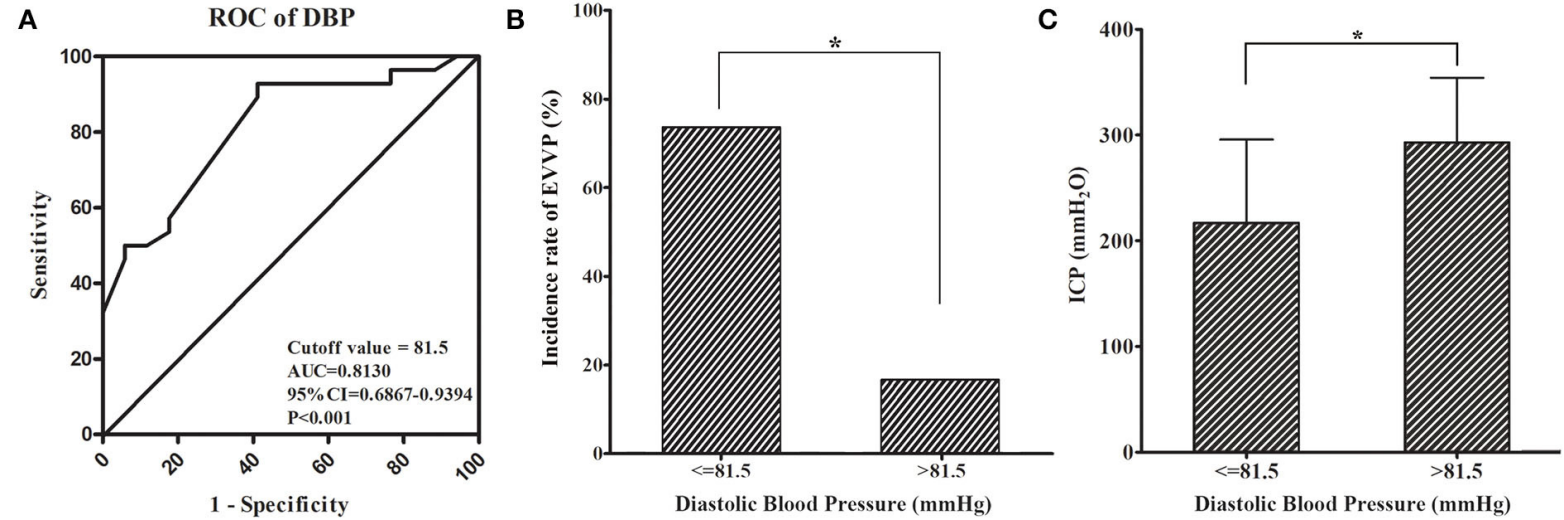
Receiver operating characteristic curve showed that DBP ≤ 81.5 mmHg indicated the presence of EVVP in BTSS patients **(A)**. The incidence of EVVP was significantly higher **(B)** and ICP was significantly lower **(C)** in BTSS patients with DBP ≤ 81.5 mmHg than those with DBP > 81.5 mmHg. * *p* < 0.05. BTSS, bilateral transverse sinus stenosis; DBP, diastolic blood pressure; EVVP, enlarged vertebral venous plexus; ICP, intracranial pressure.

In order to identify a cutoff value of DBP to predict the presence of EVVP, the ROC curve was performed. A DBP of 81.5 mmHg was calculated as the cutoff value for the presence of EVVP (AUC = 0.813, 95%CI = 0.6867–0.9394, *p* < 0.001, Figure 1A). DBP ≤ 81.5 mmHg indicated the presence of EVVP in patients with BTSS. Further analysis confirmed that the incidence of EVVP was significantly higher in patients with BTSS with DBP ≤ 81.5 mmHg than those with DBP > 81.5 mmHg (*p* = 0.002, Figure 1B). Moreover, ICP was significantly lower in patients with BTSS with DBP ≤ 81.5 mmHg than those with DBP > 81.5 mmHg (*p* = 0.008, Figure 1C).

## Discussion

As the results above have clearly demonstrated, BTSS is more prevalent in women. Miyachi et al. (9) also report a female predominance in patients with BTSS. The possible explanation is that sex hormones are involved in the pathogenesis of BTSS. However, further investigations are required to prove this hypothesis. Low percentages of smoking and alcohol rates may be due to the low proportion of male patients.

It has been reported that BTSS is correlated with IH (3). Results from our previous study also supported this finding (6). A triad of IH which manifested as headache, tinnitus, and papilledema are confirmed as the most common symptoms in patients with BTSS in this study. Moreover, hypertension has been identified as the most common comorbid disease for maintaining cerebral perfusion pressure in the presence of IH.

However, the causality between BTSS and IH has not been completely elaborated. Markey et al. (3) supported that venous sinus stenosis disturbs the reflux of cerebrospinal fluid with a resultant rise in ICP. Whereas Volpe et al. (10) yield a seemingly inconsistent result that elevated ICP compresses the venous sinus and leads to secondary venous sinus segmental stenosis. Karahalios et al. demonstrated that venous sinus stenosis still exists even if ICP has been reduced to a normal level (11). From our perspective, there may be an enhanced loop between BTSS and IH, featuring the exacerbation of either BTSS or IH while the other one emerges.

The transverse sinus-sigmoid sinus-internal jugular vein and the vertebral venous plexus are two main outflow tracts of intracranial venous blood (12). In the presence of BTSS, it is predicted that the intracranial venous blood was mainly drained through the vertebral venous plexus (13). Therefore, the presence of EVVP would guarantee sufficient cerebral venous reflux. Our previous report has demonstrated that EVVP compensates for the elevated ICP in patients with BTSS (6).

Although BMI was significantly higher in patients with BTSS without EVVP than those with EVVP, BMI was not correlated with EVVP after adjusting for DBP. It has been reported that obesity is a risk factor for idiopathic IH (14, 15). However, we found that BMI was not correlated with ICP after being adjusted for DBP. It is suggested that BMI is incapable of predicting EVVP and ICP.

In the meanwhile, we also found that DBP was significantly higher in patients with BTSS without EVVP than in those with EVVP. DBP was identified as an independent predictor of EVVP and ICP after adjusting for BMI. ROC analysis showed that DBP ≤ 81.5 mmHg indicated the presence of EVVP in patients with BTSS. Patients with BTSS with DBP ≤ 81.5 mmHg had a higher incidence of EVVP and a lower ICP compared to those with DBP > 81.5 mmHg. It is suggested that DBP ≤ 81.5 mmHg indicates a higher incidence of EVVP and therefore predicts a lower ICP in patients with BTSS.

It has been demonstrated that elevated venous pressure promotes the contraction of arteriolar smooth muscle, thus resulting in an increase in peripheral vascular resistance, which in turn leads to elevated DBP (16, 17). Therefore, lower DBP may be correlated with lower central venous pressure (CVP). The higher pressure gradient between ICP and CVP increases shear stress in the vertebral venous plexus and thereby causing EVVP. However, this hypothesis requires further verification.

The limitation of this study was the small sample size. Further validation with a larger sample size will be conducted in the future.

## Conclusion

Diastolic blood pressure was identified as an independent predictor of EVVP. DBP was lower (≤81.5 mmHg) in patients with EVVP and therefore was associated with a lower ICP in patients with BTSS.

## Data availability statement

The raw data supporting the conclusions of this article will be made available by the authors, without undue reservation.

## Ethics statement

This study was approved by the Xuanwu Hospital Ethics Committee. The patients/participants provided their written informed consent to participate in this study.

## Author contributions

ML contributed to study design, data collection, manuscript writing, and final approval of the manuscript. XG contributed to data analysis, manuscript writing, and final approval of the manuscript. FL and NX contributed to manuscript writing and final approval of the manuscript. JS contributed to data collection and final approval of the manuscript. RM contributed to acquisition of study funding, study design, critical revision of the manuscript, and final approval of the manuscript. XJ contributed to data interpretation, critical revision of the manuscript, and final approval of the manuscript. All authors contributed to the article and approved the submitted version.

## Funding

This study was supported by Beijing Natural Science Foundation (7212047). The funding agencies had no role in the design and conduct of the study; in the collection, analysis, and interpretation of the data; or in the preparation, review, or approval of the manuscript.

## Conflict of interest

The authors declare that the research was conducted in the absence of any commercial or financial relationships that could be construed as a potential conflict of interest.

## Publisher's note

All claims expressed in this article are solely those of the authors and do not necessarily represent those of their affiliated organizations, or those of the publisher, the editors and the reviewers. Any product that may be evaluated in this article, or claim that may be made by its manufacturer, is not guaranteed or endorsed by the publisher.

## References

[1] Durst CR Ornan DA Reardon MA Mehndiratta P Mukherjee S Starke RM. Prevalence of dural venous sinus stenosis and hypoplasia in a generalized population. J Neurointerv Surg. (2016) 8:1173–7. 10.1136/neurintsurg-2015-012147 26747875

[2] Bono F Lupo MR Lavano A Mangone L Fera F Pardatscher K. Cerebral MR venography of transverse sinuses in subjects with normal CSF pressure. Neurology. (2003) 61:1267–70. 10.1212/01.WNL.0000092021.88299.B4 14610135

[3] Markey KA Mollan SP Jensen RH Sinclair AJ. Understanding idiopathic intracranial hypertension: mechanisms, management, and future directions. Lancet Neurol. (2016) 15:78–91. 10.1016/S1474-4422(15)00298-7 26700907

[4] Biousse V Bruce BB Newman NJ. Update on the pathophysiology and management of idiopathic intracranial hypertension. J Neurol Neurosurg Psychiatry. (2012) 83:488–94. 10.1136/jnnp-2011-302029 22423118 PMC3544160

[5] Kelly LP Saindane AM Bruce BB Ridha MA Riggeal BD Newman NJ. Does bilateral transverse cerebral venous sinus stenosis exist in patients without increased intracranial pressure? Clin Neurol Neurosurg. (2013) 115:1215–9. 10.1016/j.clineuro.2012.11.004 23219404 PMC3610812

[6] Li M Bai C Sun J Xia N Meng R Ji X. Enlarged vertebral venous plexus alleviated intracranial hypertension-related symptoms in patients with bilateral transverse sinus stenosis. Cerebrovasc Dis. (2022) 51:525–31. 10.1159/000519716 35081531

[7] Johannesson G Eklund A Linden C. Intracranial and intraocular pressure at the lamina cribrosa: gradient effects. Curr Neurol Neurosci Rep. (2018) 18:25. 10.1007/s11910-018-0831-9 29651628 PMC5897485

[8] Lee YH Wang Z Chiang TC Liu CT. Beverage intake, smoking behavior, and alcohol consumption in contemporary China-a cross-sectional analysis from the 2011 China health and nutrition survey. Int J Environ Res Public Health. (2017) 14:493. 10.3390/ijerph14050493 28481283 PMC5451944

[9] Miyachi S Hiramatsu R Ohnishi H Takahashi K Kuroiwa T. Endovascular treatment of idiopathic intracranial hypertension with stenting of the transverse sinus stenosis. Neurointervention. (2018) 13:138–43. 10.5469/neuroint.2018.00990 30196687 PMC6132029

[10] Volpe NJ. Idiopathic intracranial hypertension: important questions answered with more to come. JAMA Neurol. (2014) 71:678–80. 10.1001/jamaneurol.2014.444 24756224

[11] Karahalios DG Rekate HL Khayata MH Apostolides PJ. Elevated intracranial venous pressure as a universal mechanism in pseudotumor cerebri of varying etiologies. Neurology. (1996) 46:198–202. 10.1212/WNL.46.1.198 8559374

[12] Jayaraman MV Boxerman JL Davis LM Haas RA Rogg JM. Incidence of extrinsic compression of the internal jugular vein in unselected patients undergoing CT angiography. AJNR Am J Neuroradiol. (2012) 33:1247–50. 10.3174/ajnr.A2953 22322614 PMC7965500

[13] Doepp F Schreiber SJ von Munster T Rademacher J Klingebiel R Valdueza JM. How does the blood leave the brain? A systematic ultrasound analysis of cerebral venous drainage patterns. Neuroradiology. (2004) 46:565–70. 10.1007/s00234-004-1213-3 15258709

[14] Pollak L Zohar E Glovinsky Y Huna-Baron R. The laboratory profile in idiopathic intracranial hypertension. Neurol Sci. (2015) 36:1189–95. 10.1007/s10072-015-2071-y 25596710

[15] Baykan B Ekizoglu E Altiokka Uzun G. An update on the pathophysiology of idiopathic intracranial hypertension alias pseudotumor cerebri. Agri. (2015) 27:63–72. 10.5505/agri.2015.22599 25944131

[16] Iida N Mitamura Y. Effects of venous pressure elevation on myogenic vasoconstrictive responses to static and dynamic arterial pressures. Jpn J Physiol. (1989) 39:811–23. 10.2170/jjphysiol.39.811 2632898

[17] Strandberg TE Pitkala K. What is the most important component of blood pressure: systolic, diastolic or pulse pressure? Curr Opin Nephrol Hypertens. (2003) 12:293–7. 10.1097/00041552-200305000-00011 12698068

